# Supplementary material for: N-Amino Peptide–Graphene Quantum Dot Loaded Small Extracellular Vesicles for Targeted Therapy of Tauopathies
Source: Adv Nanobiomed Res. Author manuscript; Available in PMC 2026 May 13. (PMC13166128; doi:10.1002/anbr.202500065)
Supplement: SI [file NIHMS2172344-supplement-SI.pdf]

Supporting Information

N-Amino Peptide-Graphene Quantum Dot Loaded Small Extracellular Vesicles for Targeted Therapy of Tauopathies

Runyao Zhu,[1] Gaeun Kim,[1] Benjamin H. Rajewski,[2] Isaac J. Angera,[2] Juan R. Del Valle,[*,2] Yichun Wang[*,1]

[1] *Department of Chemical & Biomolecular Engineering, University of Notre Dame, Indiana 46556, United States.*

[2] *Department of Chemistry & Biochemistry, University of Notre Dame, Indiana 46556, United States.*

* Corresponding author: Yichun Wang (ywang65@nd.edu); Juan R. Del Valle (jdelvalle@nd.edu)

Supporting Figures

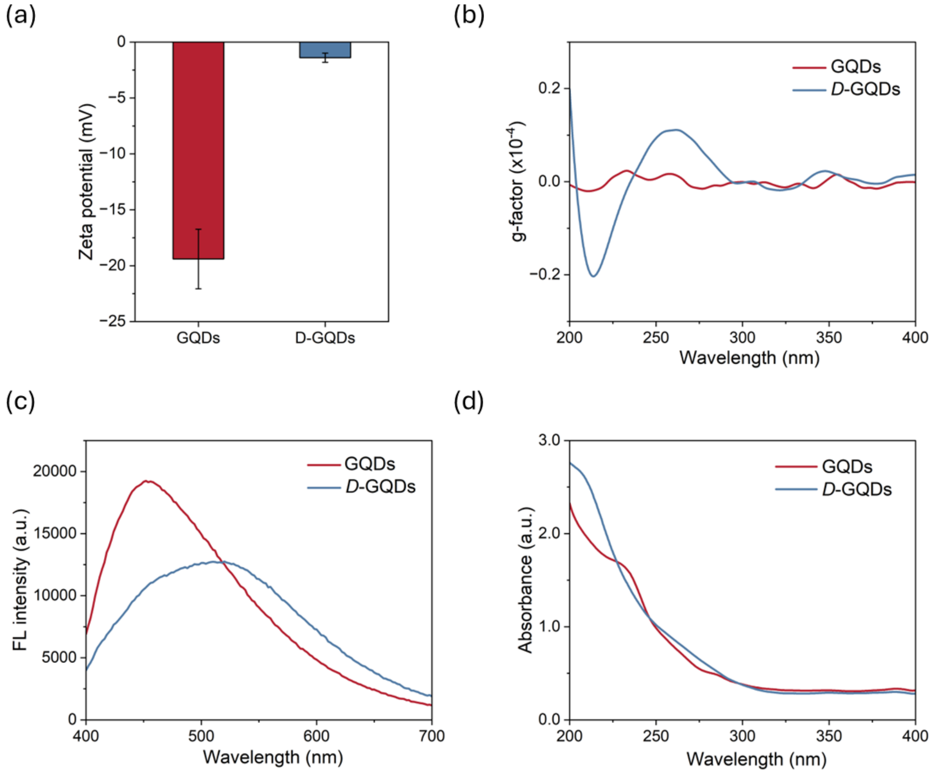

Figure S1. (a) Zeta potentials, (b) g-factor spectra, (c) fluorescence spectra (excited at 365 nm), and (d) absorbance spectra of graphene quantum dots (GQDs) and *D*-cysteine functionalized GQDs (*D*-GQDs).

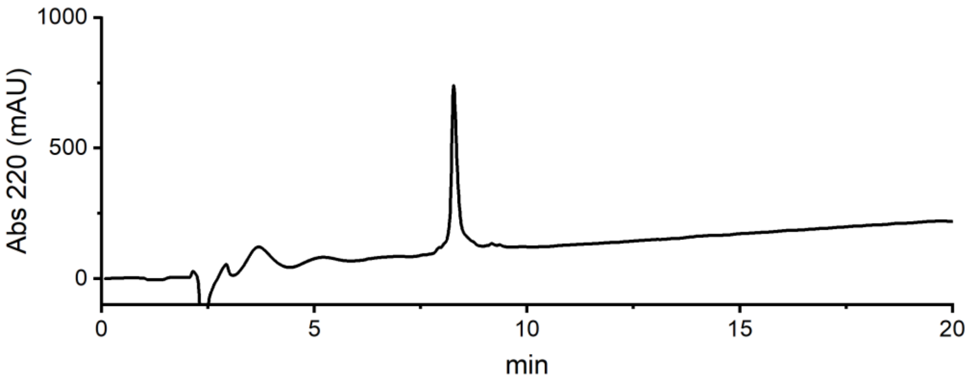

Figure S2. Mxyl-NAP2 purification and characterization. The crude peptide was purified by preparative scale RP-HPLC using a 5-80% MeCN/H_2O gradient (with 0.1% formic acid). The pure peptide was obtained in 12% overall yield based on initial resin loading. HRMS (ESI-TOF) m/z $[M + H]^+$ calcd for $C_{112}H_{174}N_{33}O_{27}S_2$ 2477.2692, found 2477.2719, err -1.1 ppm.

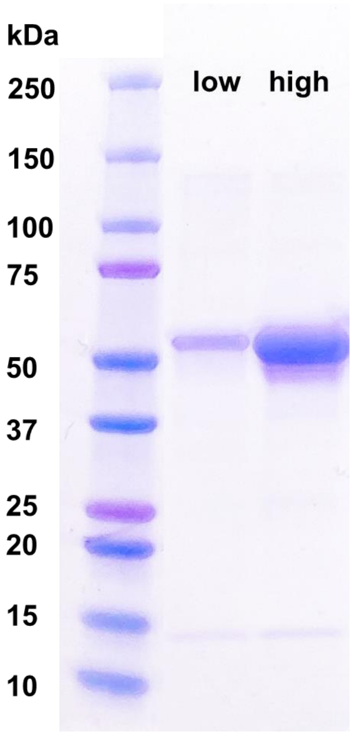

Figure S3. Sodium dodecyl sulfate-polyacrylamide gel electrophoresis (SDS/PAGE, Coomassie blue stain) of purified tau$_{P301L}$ protein loaded at low and high concentrations.

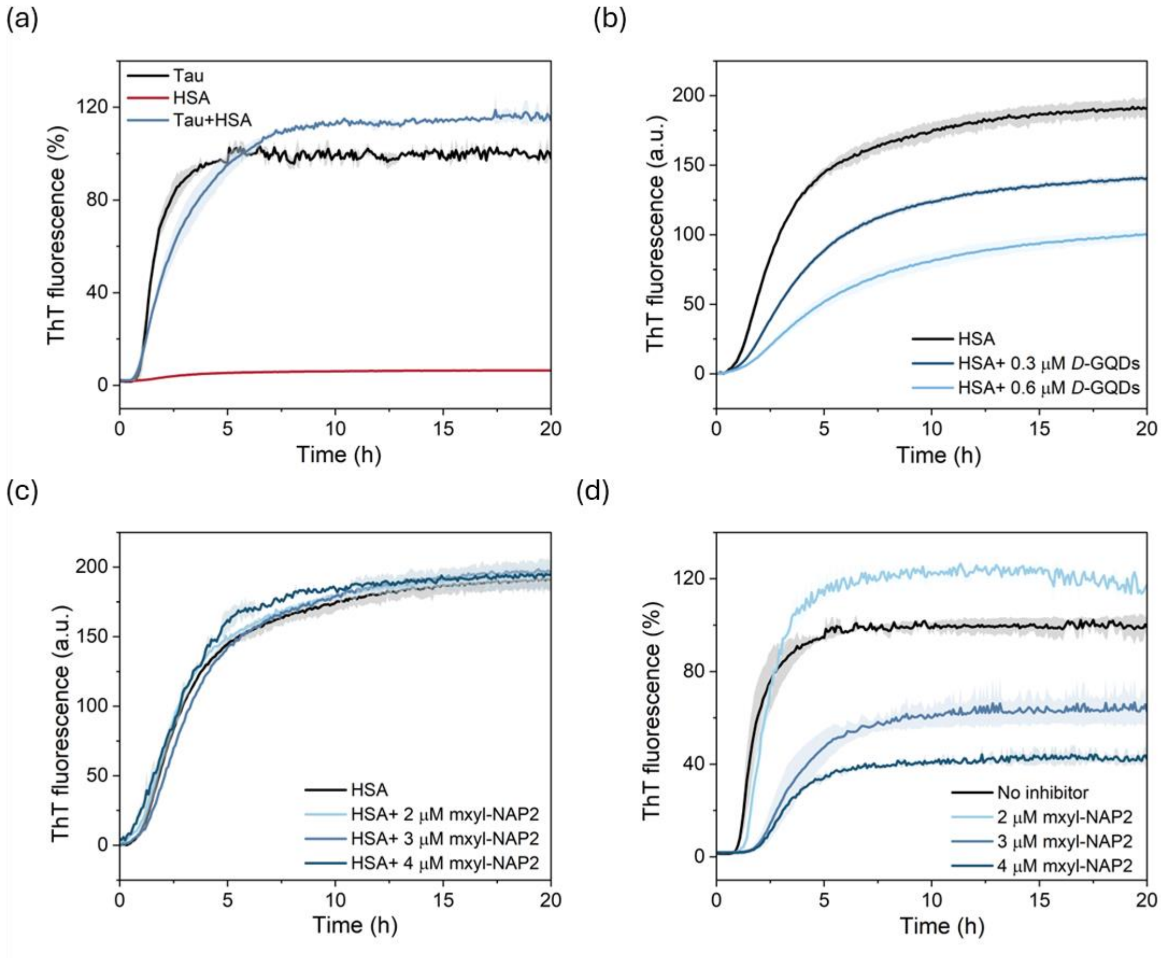

Figure S4. (a) Thioflavin T (ThT) fluorescence of tau$_{P301L}$ aggregation in the presence of human serum albumin (HSA), and tau$_{P301L}$ and HSA aggregation alone. (b-c) ThT fluorescence of HSA aggregation incubated with *D*-GQDs and mxyl-NAP2 at different concentrations. (d) ThT fluorescence of tau$_{P301L}$ aggregation incubated with different concentrations of mxyl-NAP2 (n = 3, mean ± SD).

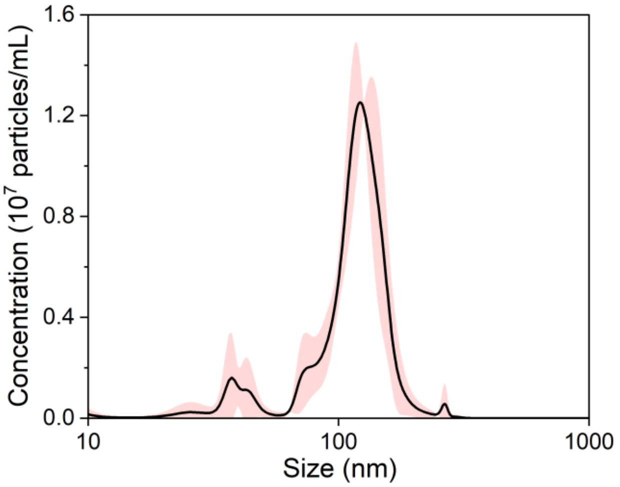

Figure S5. Nanoparticle tracking analysis (NTA) of *D*-GQD loaded small extracellular vesicles (sEVs) with an average diameter of 127.6 ± 3.1 nm.

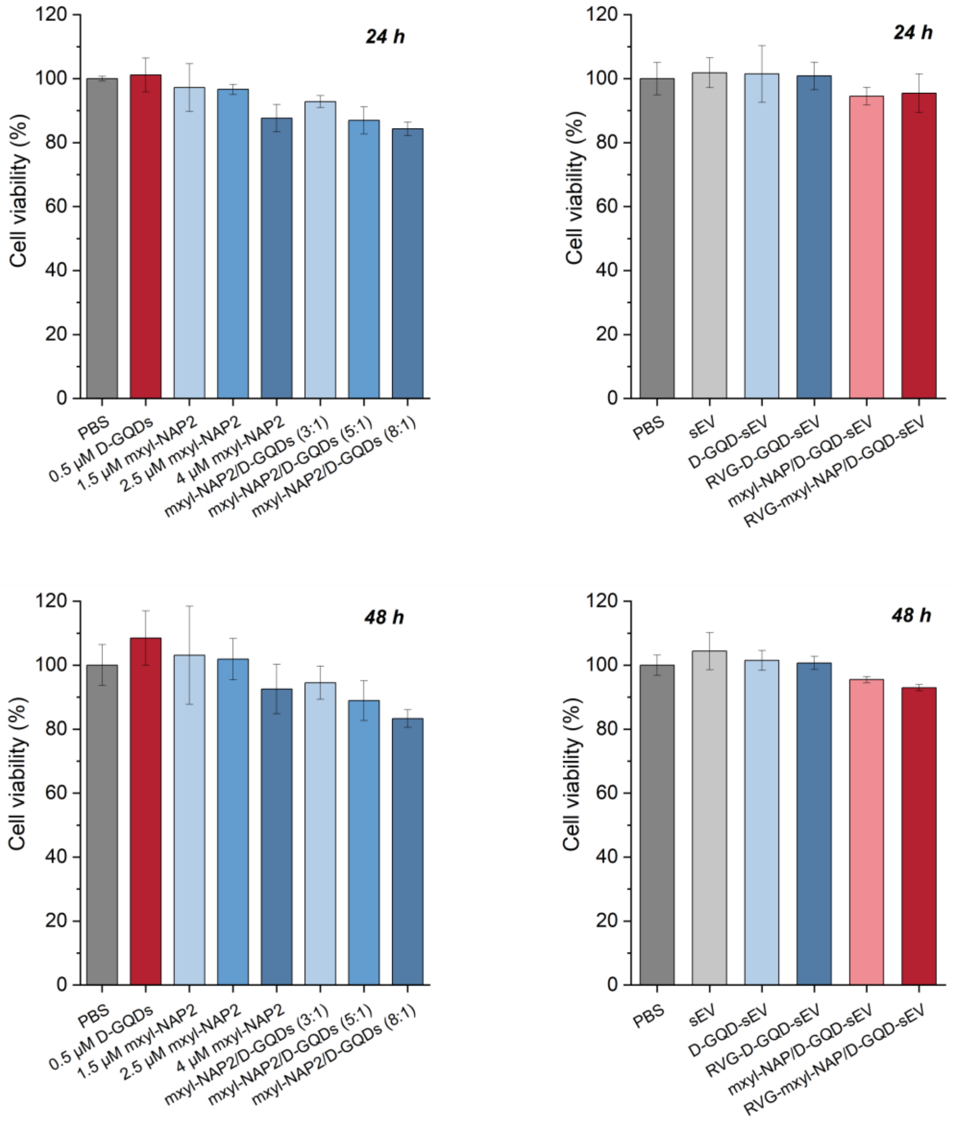

Figure S6. The cell viability of SH-SY5Y human neuroblastoma cells after incubation with various tau inhibitors, including *D*-GQDs, mxyl-NAP2, mxyl-NAP2/*D*-GQD complexes, *D*-GQD-sEV, RVG-*D*-GQD-sEV, mxyl-NAP2/*D*-GQD-sEV, and RVG-mxyl-NAP2/*D*-GQD-sEV. Cell viability was tested using CCK-8 assay after 24 and 48-h treatment (n = 3, mean ± SD). The concentration of *D*-GQDs was fixed at 0.5 μM across all inhibitors, and the molar ratio of mxyl-NAP2/*D*-GQD for sEV-encapsulated complexes was maintained at 8:1.

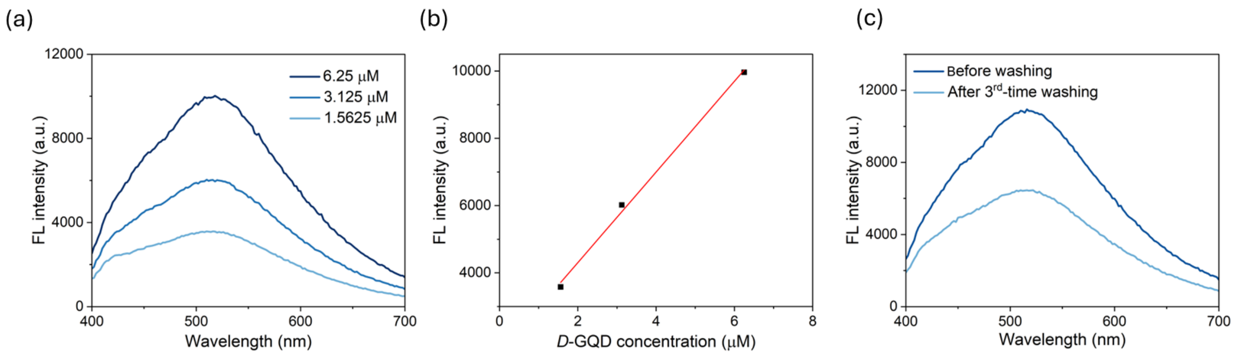

Figure S7. (a-b) The fluorescence spectra (excited at 365 nm) of *D*-GQDs at different concentrations. The fluorescence intensity at the emission peak exhibited a linear relationship with its concentration. (c) The fluorescence spectra (excited at 365 nm) of *D*-GQD loaded sEV before PBS washing and after three-time washing. Based on the concentration calibration curve of *D*-GQDs, *D*-GQD remained in the sEV solution after three-time PBS washing was 45% of the initial incubation concentration.

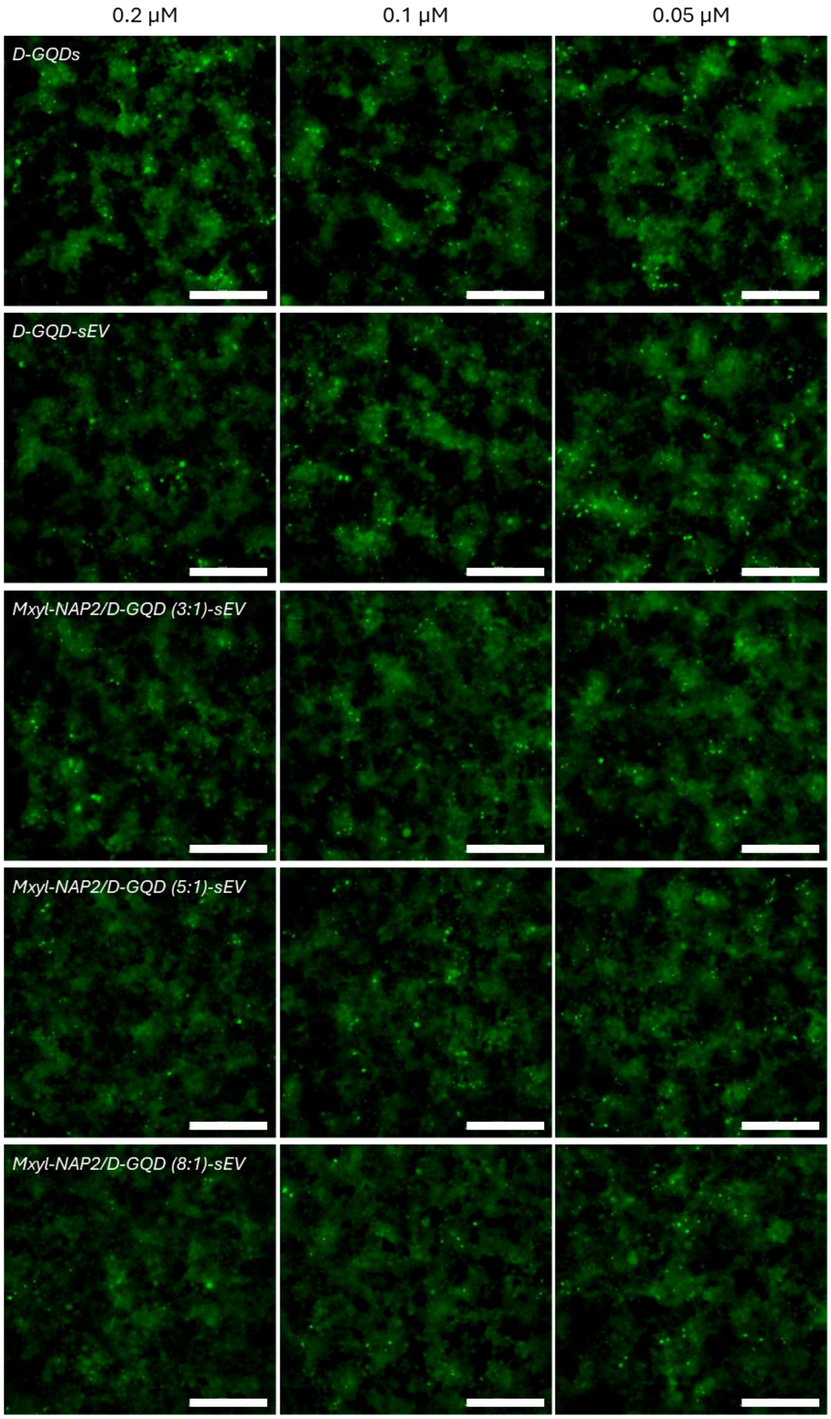

Figure S8. Mxyl-NAP2/*D*-GQD-sEVs, *D*-GQD-sEVs, and *D*-GQDs, at concentrations of 0.2, 0.1, and 0.05 μM for *D*-GQDs, sEV (1×10^9 particles/mL), prevented the cellular transmission of mature $\text{tau}_{\text{P301L}}$ fibrils (0.19 μM). Representative images of cells were taken at 20× magnification under the FITC channel (ex: 469 nm/em: 525 nm). The green puncta with high fluorescence represented the aggregation of tau in cells induced by exogenous tau fibers. Scale bar: 200 μm.

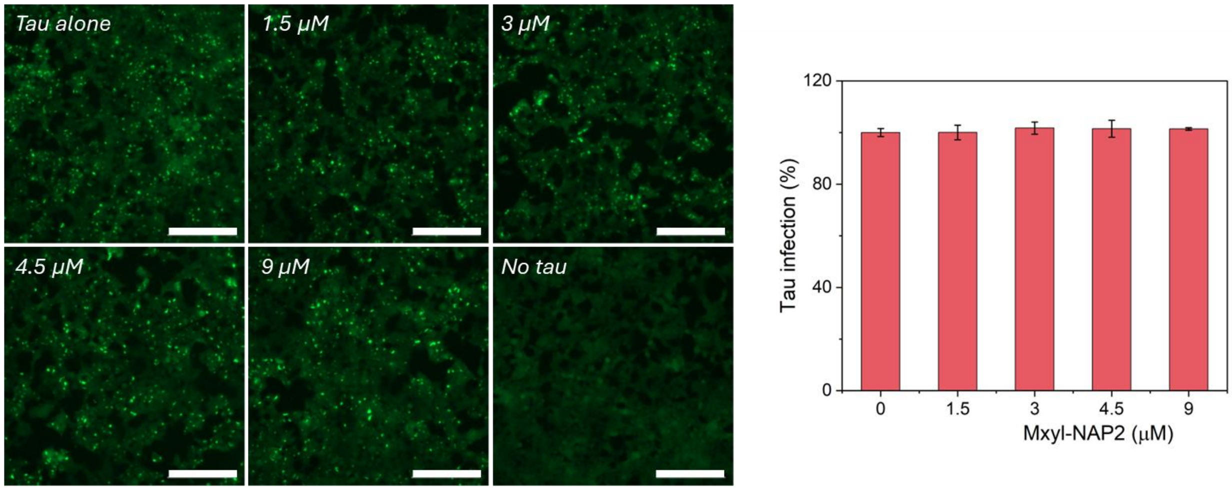

Figure S9. Mxyl-NAP2 was directly added to HEK293 cells stably expressing tau-RD (P301L/V337M)-YFP 1-h after adding tau_{P301L} fibrils and incubated for 48 h. Mxyl-NAP2 at concentrations of 1.5, 3, 4.5, and 9 μM did not show the inhibition ability on the cellular transmission of mature tau fibrils (0.19 μM). Fluorescent images of cellular tau biosensors were taken under the FITC channel (ex/em: 469/525 nm). The green puncta with high fluorescence represented the aggregation of tau in cells induced by exogenous tau_{P301L} fibers. Scale bar: 200 μm. Tau infection (%) in the bar graph shows the number of intracellular fluorescent puncta relative to control infection wells lacking the inhibitors (n = 3, mean ± SD).

.

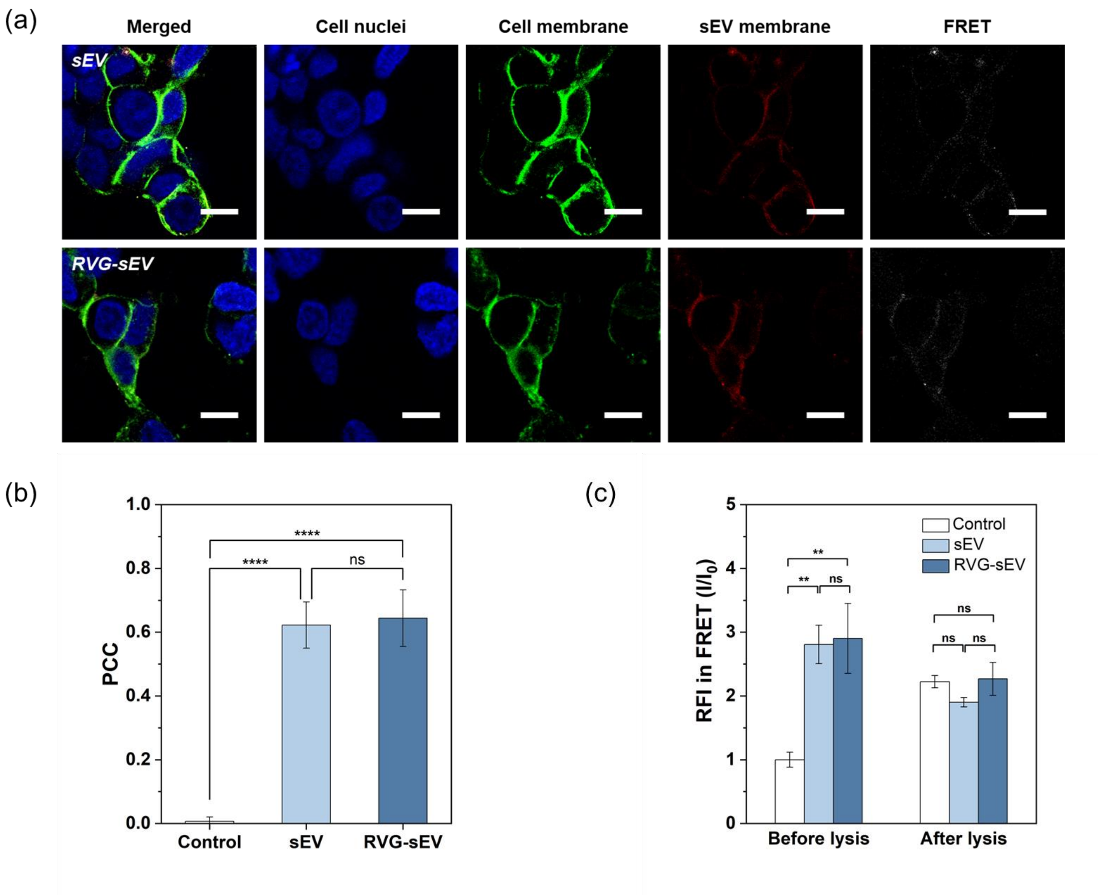

Figure S10. (a) CLSM images of SH-SY5Y cells incubated with *D*-GQD-sEVs and RVG-*D*-GQD-sEVs for 1 h. Each channel represents: blue for nuclei, green for cellular membrane, red for sEV membrane, and gray for FRET. Scale bar: 20 μm. (b) PCC was quantitatively analyzed based on merged CLSM images (n = 6, mean ± SD). (c) FRET evaluation by measuring fluorescence intensity (FI) in a cell suspension (ex, 488 nm; em, 580 nm) (n = 3, mean ± SD). The control group was SH-SY5Y cells incubated in the cell culture medium without sEV treatment. *P*-values are calculated using unpaired two-sided Student's *t-test*, *ns*: not significant, *p < 0.05, **p < 0.01, ****p < 0.0001.

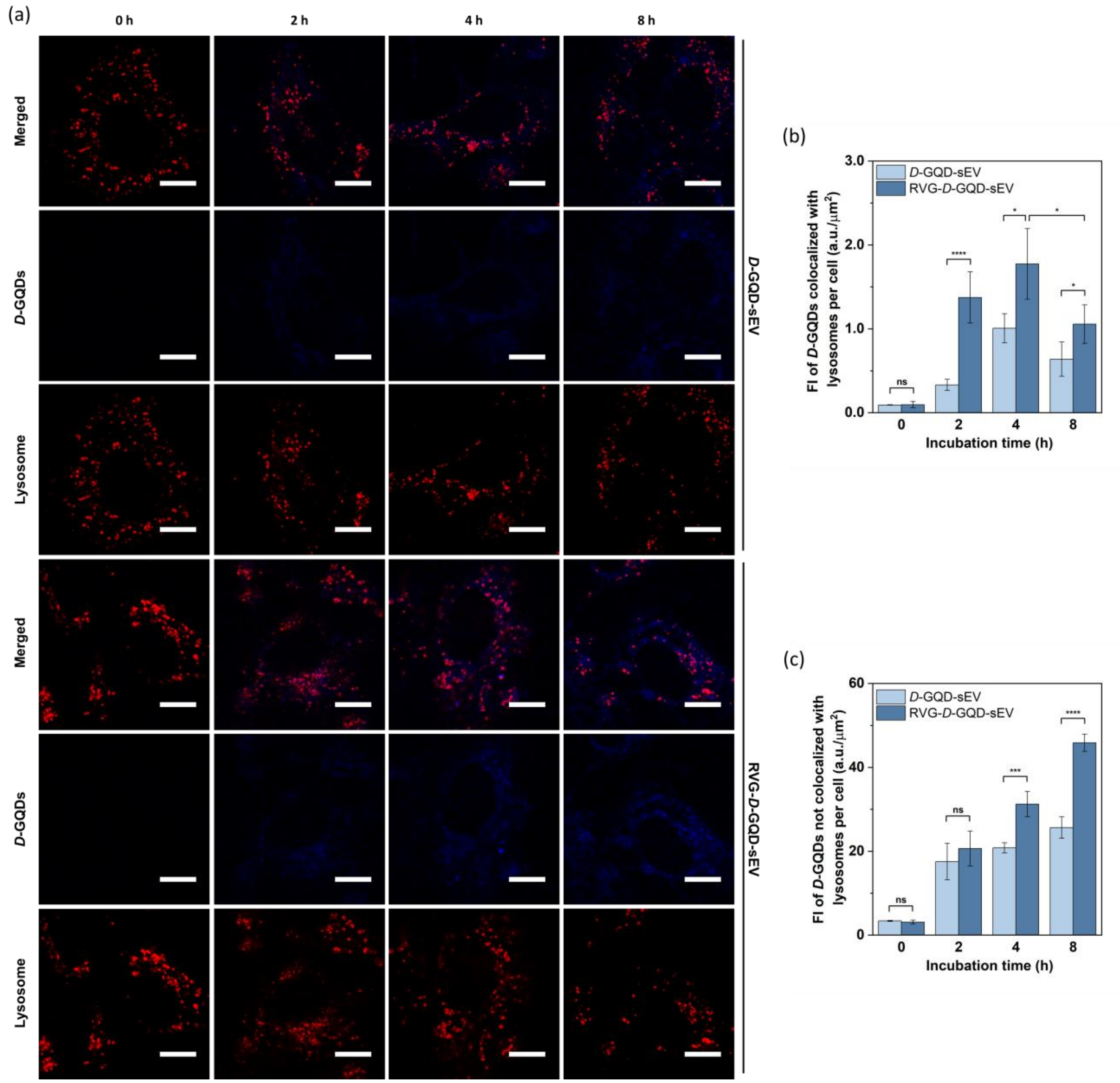

Figure S11. Time-dependent colocalization analysis of *D*-GQDs with lysosomes. (a) CLSM images of SH-SY5Y cells incubated with *D*-GQD-sEVs and RVG-*D*-GQD-sEVs for 0, 2, 4, and 8 h. Each channel represents: blue for *D*-GQDs and red for lysosomes. Scale bar: 10 μm. The quantification of *D*-GQDs FI (b) colocalized with lysosomes and (c) outside of lysosomes per cell (n = 4, mean ± SD). *P*-values are calculated using unpaired two-sided Student's *t-test*, *ns*: not significant, *$p < 0.05$, ***$p < 0.001$, ****$p < 0.0001$.

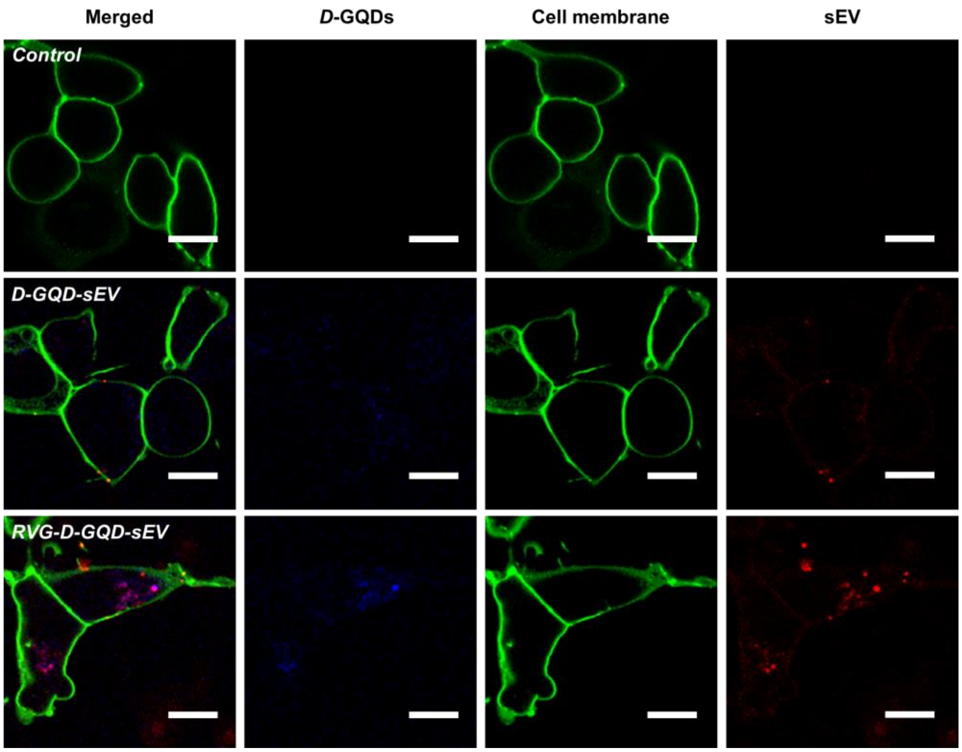

Figure S12. *D*-GQDs release and retention from sEVs after SH-SY5Y cells incubated with *D*-GQD-sEVs and RVG-*D*-GQD-sEVs for 2 h. Each channel represents: blue for *D*-GQDs, green for the cellular membrane, and red for the sEV membrane. Scale bar: 10 μm. The control group was SH-SY5Y cells incubated in the cell culture medium without sEV treatment.

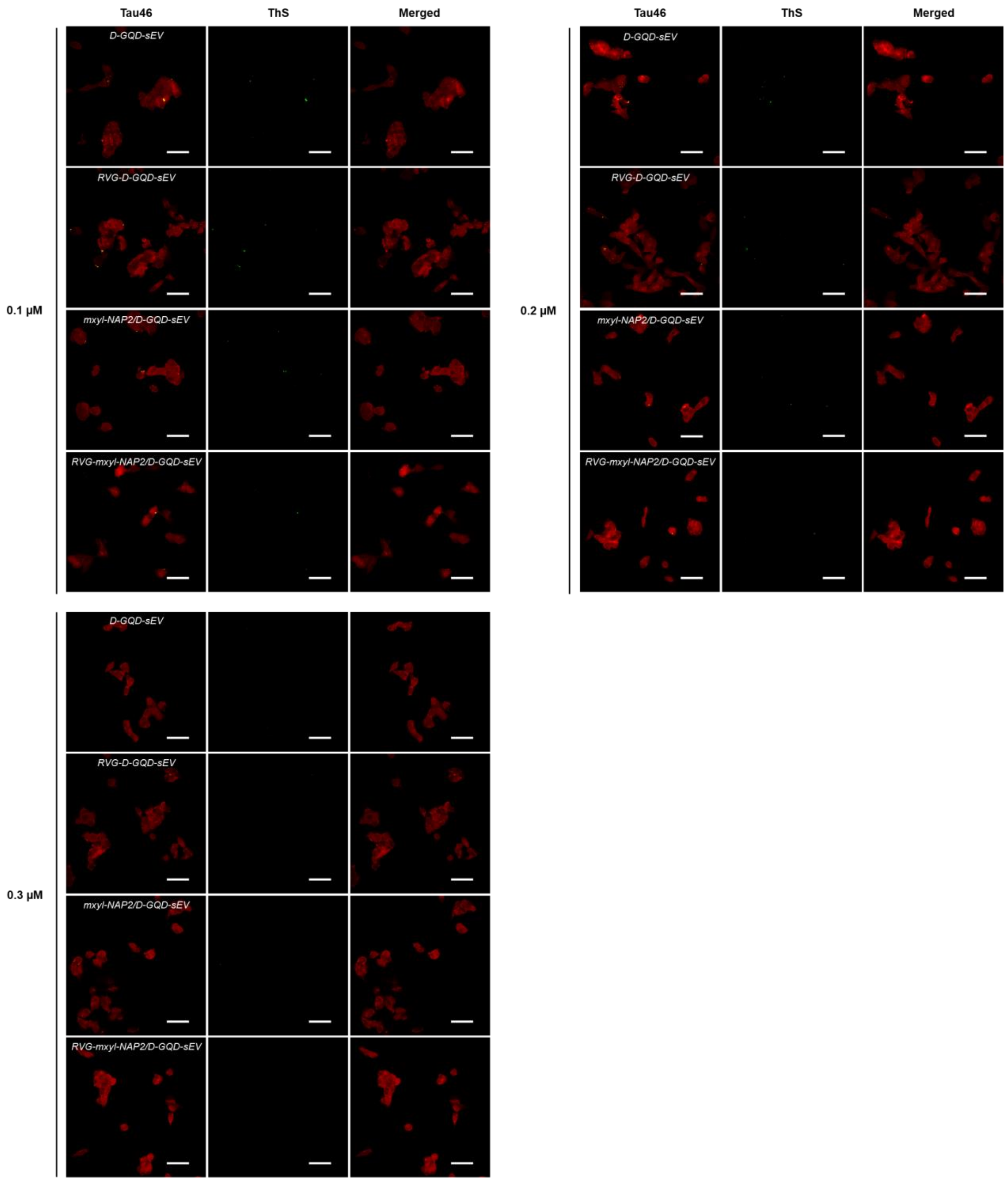

Figure S13. CLSM images of tau fibril-treated SH-SY5Y cells incubated with different inhibitors (*D*-GQD-sEV, RVG-*D*-GQD-sEV, mxyl-NAP2/*D*-GQD-sEV, and RVG-mxyl-NAP2/*D*-GQD-sEV) at concentrations of 0.1, 0.2, and 0.3 μM for *D*-GQDs. SH-SY5Y cells were sequentially stained with antibody Tau46 (red) for intracellular tau and Thioflavin S (ThS, green) for tau fibrils. Scale bar: 50 μm.

Supporting Tables

Table S1. Encapsulation efficiency of mxyl-NAP2 in sEV with different ratios of mxyl-NAP2 to *D*-GQDs was determined by the absorbance at 292 nm of encapsulated mxyl-NAP2 which was separated from *D*-GQDs and sEV membrane after lysis.

	Abs at 292 nm	Concentration of mxyl-NAP2	Encapsulation efficiency
Mxyl-NAP2/*D*-GQD (8:1)-sEV	1.0336	24.67 μM	30.8%
Mxyl-NAP2/*D*-GQD (5:1)-sEV	1.0299	18.50 μM	38.5%
Mxyl-NAP2/*D*-GQD (3:1)-sEV	1.0249	10.08 μM	42.0%
